# Gaze cueing, mental States, and the effect of autistic traits

**DOI:** 10.3758/s13414-021-02368-0

**Published:** 2021-09-14

**Authors:** Emma J. Morgan, Daniel T. Smith, Megan Freeth

**Affiliations:** 1 Psychology Department, University of Sheffield Cathedral Court, 1 Vicar Lane, Sheffield, S1 2LT England; 2 Psychology Department, Durham University, Upper Mountjoy, South Road, Durham, DH1 3LE England

**Keywords:** Gaze cueing, Social attention, Autistic traits, Mental states, Theory of mind

## Abstract

The ability to interpret and follow the gaze of our social partners is an integral skill in human communication. Recent research has demonstrated that gaze following behaviour is influenced by theory of mind (ToM) processes. However, it has yet to be determined whether the modulation of gaze cueing by ToM is affected by individual differences, such as autistic traits. The aim of this experiment was to establish whether autistic traits in neurotypical populations affect the mediation of gaze cueing by ToM processes. This study used a gaze cueing paradigm within a change detection task. Participants’ perception of a gaze cue was manipulated such that they only believed the cue to be able to ‘see’ in one condition. The results revealed that participants in the Low Autistic Traits group were significantly influenced by the mental state of the gaze cue and were more accurate on valid trials when they believed the cue could ‘see’. By contrast, participants in the High Autistic Traits group were also more accurate on valid trials, but this was not influenced by the mental state of the gaze cue. This study therefore provides evidence that autistic traits influence the extent to which mental state attributions modulate social attention in neurotypical adults.

Attending to the gaze direction of others is a fundamental building block of human communication and social cognition. For neurotypical individuals, the mere perception of another person’s gaze direction is sufficient to drive a shift in attention (Driver et al., 1999; Ristic & Kingstone, 2005), and recent research has demonstrated that this attentional shift is likely modulated by theory of mind processes (Gobel & Giesbrecht, 2020; Morgan et al., 2018). However, autistic individuals often show difficulties with theory of mind processes (Baron-Cohen et al., 2001), atypical attentional responses to eye-gaze direction (Freeth & Bugembe, 2019; Freeth et al., 2010), and somewhat reduced accuracy in making line-of-sight judgements (Freeth et al., 2020; Pantelis & Kennedy, 2017). Individuals within the neurotypical population can also demonstrate traits associated with the Broad Autism Phenotype (Hurley et al., 2007), and there has been some recent suggestion that autistic traits influence gaze cueing task performance involving emotion processing (de Araújo et al., 2021). However, it is yet to be determined whether autistic traits influence the extent to which mental state attributions mediate gaze cueing in neurotypical individuals.

Neurotypical individuals display a remarkably clear preference to attend to the eyes in a face, starting within their first 24 hours of life (Farroni et al., 2002) and continuing into adulthood (Levy et al., 2013). The early preference to attend to the eyes develops into an ability to follow gaze direction by 3 months of age, and an ability to orient our attention to the end location of a gaze cue by 12 months of age (Dalmaso et al., 2020). The ability to follow a gaze cue is argued to be an essential skill in allowing us to follow and engage in social interactions, with the gaze direction of our social partners acting as a signal to important events and objects within our environment (Capozzi & Ristic, 2018). Indeed, the influence of a social partner’s gaze direction on our own attention is clearly demonstrated via the gaze cueing effect (Driver et al., 1999). In typical gaze cueing paradigms, a participant demonstrates significantly faster reaction times to detect a target when the location of the target is validly indicated by the eye direction of a stimulus face (Driver et al., 1999; Ristic & Kingstone, 2005). The gaze cueing effect is a robust phenomenon that occurs rapidly in response to viewing the eye movements of another person, and its existence has been demonstrated consistently throughout the period in which it has been studied. Therefore, the gaze of others has been proven to be important not only in terms of its social relevance but also in terms of its ability to orient our attention to key information within our environment (Dalmaso et al., 2020).

However, the processes underlying gaze cueing are strongly contested. Gaze cueing is often argued to be a reflexive process which occurs rapidly and outside of the influence of conscious, top-down processing (Cole et al., 2017; Cole et al., 2015). Yet, in contrast to this claim, several studies have provided key evidence that gaze cueing can be guided by top-down attributions, even at time latencies associated with bottom-up, spontaneous processing (Dalmaso et al., 2014; Gobel & Giesbrecht, 2020; Morgan et al., 2018). Gaze cueing has been proven to be guided by the top-down influence of relevant social information—for example, the social status, emotional valance, or age of our social partner (Dalmaso, et al., 2020; Gobel & Giesbrecht, 2020). However, of key consideration to this study, it is argued that our beliefs regarding the mental states of our social partners also play a crucial role in influencing the extent to which we follow their gaze (Baker et al., 2016; Furlanetto et al., 2016; Morgan et al., 2018; Teufel et al., 2010). Indeed, in a recent study, we replicated previous research (Nuku & Bekkering, 2008; Teufel et al., 2010) demonstrating that participants showed significantly reduced gaze cueing effects if they believed a social partner to be unable to physically ‘see’ a target (Morgan et al., 2018). This therefore suggests that whilst gaze cueing may occur rapidly and automatically, it is still open to influence by top-down processing, such as when we attribute mental states to our social partners.

Autism Spectrum Conditions (ASC) are characterized by difficulties in social communication and social interactions across multiple contexts, including deficits in nonverbal behaviours such as eye contact (*DSM-5*; America Psychiatric Association, 2013). Of key interest, as a spectrum condition, individuals within the neurotypical population can also demonstrate traits associated with the Broad Autistic Phenotype. Such individuals may display characteristics associated with a diagnosis of an ASC, and yet remain below the clinical cut-off for diagnosis (Hurley et al., 2007). Autistic individuals show consistent differences to neurotypical individuals in their attention to the eye region of a face, with autistic individuals demonstrating a stronger preference to attend to the mouth rather than the eyes (Hanley et al., 2015). Leading from this, research has suggested that autistic individuals may not show a gaze cueing effect (Riby et al., 2013; Wykowska et al., 2015), and other research suggests that higher levels of autistics traits in neurotypical individuals can lead to reduced gaze cueing effects (Alwall et al., 2010; Bayliss & Tipper, 2005; Lin et al., 2020). Further, autistic individuals have also been found to consistently experience difficulties with theory mind processing and difficulties with assigning mental states to their social partners (Abell et al., 2000). Likewise, neurotypical individuals with high levels of autistic traits also perform significantly worse on perspective taking and theory of mind tasks than participants with low levels of autistic traits (Gökçen et al., 2014; Gökçen et al., 2016; Lockwood et al., 2013). However, despite this research, and the high prevalence of autistic traits within the general population, to date there is still little understanding of the relevance of autistic traits to the impact of theory of mind processes on the gaze cueing effect.

The aim of the current study was therefore to investigate the impact of autistic traits on the mediation of the gaze cueing effect by mental state attributions. The current study was a replication of our previous study, which investigated the influence of mental state attributions on the gaze cueing effect in neurotypical adults (Morgan et al., 2018); however, in this study, whether individuals were high or low in autistic traits was considered as an additional factor. The study used a change detection paradigm within a gaze cueing task; participants were presented with an array of four symbols and a centrally presented face. The face was wearing either yellow or red sunglasses, and participants were informed that one of these colours indicated that the agent was unable to ‘see’ through the lenses of the sunglasses. Participants were asked to complete the change detection paradigm and indicate if one of the four symbols had changed between an initial presentation and a subsequent presentation. The face could either validly cue the location of the change (gaze at the symbol that would change) or invalidly cue the location of the change (gaze to another location). Two stimulus onset asynchronies (SOAs) were used; a short SOA associated with reflexive processing and a longer SOA associated with top-down processing. The study found that participants demonstrated a gaze cueing effect, and that this effect was modulated by the mental state attribution of ‘seeing’ at both the short and long SOA, with participants following the gaze cue more when they believed it to be able to ‘see’. In the current study all participants completed a measure of autistic traits and were consequently divided into two groups based on their total scores. In line with previous studies, individuals were assigned to a group via a median-split; those who scored above the median were assigned to the High Autistic Traits group, and those who scored below the median were assigned to the Low Autistic Traits group (Alink & Charest, 2020; O’Keefe & Lindell, 2013; Vabalas & Freeth, 2016). Based on previous research indicating that the presence of autistic traits can lead to difficulties with theory of mind abilities (Gökçen et al., 2014; Gökçen et al., 2016; Lockwood et al., 2013) we predicted that for the participants with high amounts of autistic traits, the gaze cueing effect would not be influenced by the mental state of a gaze cue agent. Conversely, for the participants with low amounts of autistic traits we predict that the gaze cueing effect will be influenced by the mental state of a gaze cue agent.

## Materials and methods

### Participants

An a priori power analysis revealed that on the basis of the effect size observed in the original study (Morgan et al., 2018; *f* = 0.18), a minimum of 31 participants would be needed in each group in order to detect a significant effect of *α* = 0.05 with statistical power to detect such an effect with 80% probability. Seventy-five participants (52 female and 17 male), with a mean age of 19.65 years (range: 18–29 years, *SD* = 2.40) were recruited via opportunity sampling from an undergraduate cohort, receiving course credit for taking part. The study was approved by the Department of Psychology Ethics Committee, and all participants gave informed consent before participating. All participants had normal, or corrected-to-normal, vision. Additionally, all participants completed the Broad Autism Phenotype Questionnaire (BAPQ), a self-report questionnaire designed to measure the number of autistic traits present in a neurotypical population. Nine participants had a high rate of reporting false positives on the catch trials and were excluded from the final analysis, leaving a final sample of 66 participants. Based on a median split (med = 94) of participants’ total scores on the BAPQ, participants were divided into two groups: those high in autistic traits and those low in autistic traits (see Table 1).

**Table 1:** Participant characteristics

	High Autistic Traits	Low Autistic Traits
Gender (Male : Female)	9 : 24	8 : 25
Age		
Mean	19.30	19.47
*SD*	2.10	1.98
Range	18-29	18-24
BAPQ		
Mean	115.41**	81.18**
*SD*	17.99	11.30
Range	96-182	54-94

*Note.* BAPQ = Broad Autism Phenotype Questionnaire. ** denotes significant between group difference, *p <* .001.

### Design

The study used a mixed-model design with four independent variables: condition (seeing or nonseeing), validity (valid or invalid), group (low or high autistic traits) and stimulus onset asynchrony (SOA; 230 ms or 1,080 ms). The use of two SOAs allowed a measure of early processing and later top-down effects. The experimental trials were randomized across condition, validity, and SOA. The study paradigm was a change detection task, which required participants to correctly identify whether one of four symbols (displayed in each corner of the screen) had changed. The study was preregistered on the Open Science Framework (osf.io/cxyq4).

### Materials and apparatus

**Fig. 1:**
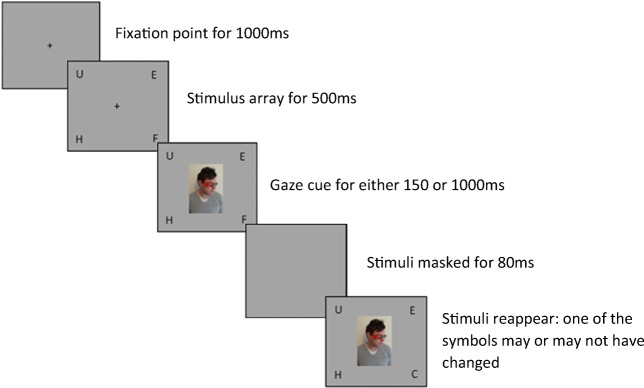
The experimental procedure. Trial types were randomized based on validity, condition, and stimulus onset asynchrony (SOA). The figure illustrates a valid trial

The study used the same change detection task as used by Morgan et al. (2018). In this paradigm, participants viewed photographs of an actor wearing a pair of either red or yellow sunglasses. Participants were informed that the actor was only able to see whilst wearing one of the pairs of sunglasses, with the colour of the ‘seeing’ sunglasses counterbalanced between participants. The gaze cue agent was centrally presented and appeared to gaze at one of four probe stimuli presented in the four corners of the screen (see Fig. 1). Each photograph used the same actor, and the stimuli for each condition differed only on the colour of the sunglasses used. The probe stimuli could be either E, U, O, P, S, F, H, L, or A and measured 1.8 × 1.8 cm. The probe stimuli appeared 5 cm away from the initial fixation point.

All participants also completed the Broad Autism Phenotype Questionnaire (BAPQ). This 36-item questionnaire was chosen for use as it is designed to be sensitive to the broader autism phenotypes present within neurotypical populations (Hurley et al., 2007). The BAPQ has demonstrated a high sensitivity (>70%) to detecting these phenotypes, and therefore was suitable for use in this study as a measure of the number of autistic traits present in the neurotypical participants who took part in the study.

### Procedure

Prior to commencing the main part of the study, all participants first completed the BAPQ. The procedure for the main experiment followed the procedure used in Morgan et al. (2018). Each participant first completed 10 practice trials, on which they were required to achieve 50% accuracy to progress to the main experiment. If participants did not meet 50% accuracy on their first attempt they were able to retake the practice trials twice more. During the main experiment, each participant completed three blocks of 80 trials, completing 240 trials in total. The study had 20% valid trials, 60% invalid trials, and 20% catch trials, in which no change occurred. As there were four potential stimulus locations, a 4:1 ratio of valid to invalid trials was necessary to ensure that the gaze cue was nonpredictive of change location.

Prior to starting the main experiment participants were shown two brief videos, each approximately 15 s in length (https://osf.io/hydfc/). The videos were designed to instil the concept that the actor could ‘see’ whilst wearing one of the pairs of coloured sunglasses and could not ‘see’ whilst wearing the other. It was emphasized that the direction in which the actor faced was nonpredictive and would not indicate where the change would occur.

Trials began with the onset of a fixation point, which was present for 1,000 ms. This was replaced with the stimulus array containing four letters, each placed in one of the four corners of the screen for 500 ms. The cue was present for either 150 ms or 1,000 ms. The display was then masked for 80 ms, after which the screen refreshed to a new display of the stimulus head and four symbols (see Fig. 1).

The participant was then required to press either ‘B’ or ‘N’ on their computer keyboard to indicate whether any of the four symbols had changed. The participant pressed ‘B’ if they believed one of the symbols had changed, and ‘N’ if they believed none had changed.

## Results

A 2 × 2 × 2 x 2 mixed-model analysis of variance (ANOVA), with three within-subject factors of condition (seeing/nonseeing), SOA (short/long), and validity (valid/invalid), and one between-subjects factor of group (high autistic traits/low autistic traits) on the probability of correctly identifying a change revealed a main effect of validity, *F*(1, 64) = 13.71, *p <* .001, *ηρ*^*2*^ *=* .18, as the proportion of correct responses was greater for the valid trials, and a main effect of SOA, *F*(1, 64) = 75.61, *p <* .001, *ηρ*^*2*^ *=* 0.54, as the proportion of correct responses for was greater for the long SOA. Critically, there was a significant Validity × Condition × Group interaction, *F*(1, 64) = 6.98, *p =* .010, *ηρ*^*2*^ *=* .10, and there was also a significant Condition × SOA × Group interaction, *F*(1, 64) = 5.06, *p =* .028, *ηρ*^*2*^ *=* .07, indicating that group membership had an impact on task performance, the nature of which is explored in the following two sections. There was no Condition × SOA × Validity × Group interaction, *F*(1, 64) = 1.35, *p = .*249, *ηρ*^*2*^ *=* .02, and no SOA × Validity × Condition interaction, *F*(1, 64) = 0.41, *p =* .523, *ηρ*^*2*^ *=* .01, indicating that the nature of the Validity × Condition interaction did not differ between the Long and Short SOA. This demonstrates that SOA did not influence the extent to which the gaze cueing effect was mediated by the mental state attribution.

### Mental state attributions: ANOVA

In order to investigate the critical Validity × Condition x Group interaction, two separate 2 × 2 repeated-measures ANOVAs were conducted, one for the High Autistic Traits group and one for the Low Autistic Traits groups. The analysis revealed that both the Low Autistic Traits group, *F*(1, 33) = 5.14, *p = .*030, *ηρ*^*2*^ *= .*14, and High Autistic Traits group, *F*(1, 33) = 9.57, *p* = .004, *ηρ*^*2*^ *= .*24, had a significant main effect of validity; both groups were more accurate on the valid trials (High Traits *M* = .80; Low Traits *M* = .77) , than on the invalid trials (High Traits *M* = .76; Low Traits *M* = .73) and therefore both groups showed a gaze cueing effect.

**Fig. 2:**
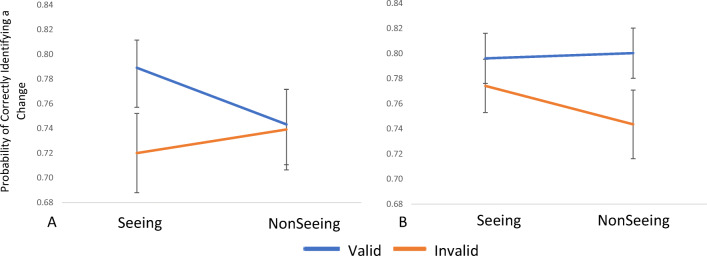
The probability of correctly identifying a change on the valid and invalid trials in the Seeing and Nonseeing conditions for the Low Autistic Traits group (**a**) and the High Autistic Traits group (**b**). Error bars show ±1 within-subject standard error of the mean (*SEM*)

However, while the Low Autistic Traits group showed a significant Condition × Validity interaction, *F*(1, 33) = 4.62, *p = .*039, *ηρ*^*2*^ *= .*12, the High Autistic Traits group did not show the same interaction, *F*(1, 31) = 2.51, *p = .*123, *ηρ*^*2*^ *= .*08. Bonferroni-corrected post hoc paired-samples *t* tests therefore investigated the interaction present in the Low Autistic Traits group. The analysis revealed that participants demonstrated a gaze cueing effect in the seeing condition and were significantly more likely to detect a change in the seeing condition when the cue was valid (*M* = 0.79, *SD* = 0.13), compared to when the cue was invalid (*M* = 0.72, *SD* = 0.17), *t*(33) = 2.72, *p* = .010. By contrast, in the nonseeing condition the same participants did not display this cueing effect and there was no significant difference between the valid (*M* = 0.74, *SD* = 0.19) and invalid trials (*M* = 0.74, *SD* = 0.19), *t*(33) = 0.25, *p =* .807*.* These results demonstrate that participants in the Low Autistic Traits group only were influenced by the mental state of the cue-agent as when the cue-agent could see, validly cued targets were more likely to be detected than invalidly cued targets. However, when the cue-agent could not see, validly cued targets were no more likely to be detected than invalidly cued targets (see Fig. 2).

### Mental state attributions: Regression

Further exploratory analyses were conducted to assess whether treating autistic traits as a continuous, rather than dichotomous, variable would also lead to the conclusion that the gaze cueing effect in those higher in autistic traits was less influenced by the mental state of the cue-agent.

Two difference scores were calculated by subtracting the invalid-seeing trials from the valid-seeing trials, and the invalid-nonseeing trials from the valid-nonseeing trials. These scores reflected the strength of the gaze cueing effect for each participant in the seeing and nonseeing conditions. For the seeing condition, the regression analysis was not significant, with participants’ total score on the BAPQ accounting for 0% of the variance in the gaze cueing effect in the Seeing condition, *F*(1, 64) = .03, *p* = .867. This demonstrates that in the seeing condition, the gaze cueing effect was not affected by the number of autistic traits each participant possessed.

By contrast, in the nonseeing condition Pearson’s correlations indicated a significant positive relationship between participants’ total score on the BAPQ and the strength of the gaze cueing effect on nonseeing trials (*r* = .34, *p* = .006). A linear regression confirmed that there was a significant relationship between BAPQ scores and the gaze cueing effect in the nonseeing condition; BAPQ scores accounted for 11.2% of the variance in the gaze cueing effect in the nonseeing condition, *F*(1, 64) = 8.07, *p* = .006. Therefore, higher amounts of autistic traits were associated with stronger gaze cueing effects in the nonseeing condition, this confirms that individuals with higher amounts of autistic traits were less influenced by the mental state of the cue-agent and showed a gaze cueing effect regardless of whether the cue-agent could see the target. This analysis therefore replicates the results of the ANOVA analysis and confirms that autistic traits influence the extent to which mental state attributions affect the gaze cueing effect.

### Stimulus onset asynchrony

**Fig. 3:**
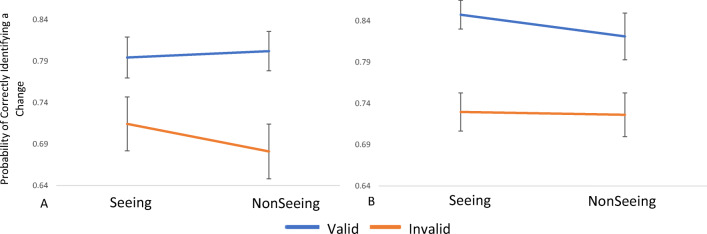
The probability of correctly identifying a change at the Long and Short SOA in the Seeing and Nonseeing conditions for the Low Autistic Traits group (**a**) and the High Autistic Traits group (**b**). Error bars show ±1 within-subject standard error of the mean (*SEM*)

The 2 × 2 × 2 × 2 mixed-model ANOVA revealed a significant Condition × SOA × Group interaction. To further investigate this interaction, two separate 2 × 2 repeated-measures ANOVAs were conducted for the High and Low Autistic Traits groups. The analysis revealed that both the Low Autistic Traits group, *F*(1, 33) = 35.82, *p < .*001, *ηρ*^2^ = .52, and High Autistic Traits group, *F*(1, 33) = 9.57, *p* = .004, ηρ^2^ = .24, had a significant main effect of SOA; both groups were more accurate at the Long SOA (Low Traits *M* = .80; High Traits *M* = .83) compared to the Short SOA (Low Traits *M* =.70; High Traits *M* = .73; see Fig. 3).

For the Low Autistic Traits group there was also a marginally significant Condition × SOA interaction, *F*(1, 33) = 4.13, *p = .*050, *ηρ*^2^ = .11, whilst the High Autistic Traits group did not show the same interaction, *F*(1, 31) = 1.35, *p = .*255, *ηρ*^*2*^ *= .*04. Bonferroni corrected post hoc paired-samples *t* tests revealed that in the Seeing condition, the Low Autistic Traits group were significantly more likely to detect a change at the Long SOA (*M* = .79, *SD* = .14) compared with the Short SOA (*M* = .71, *SD* = .14), *t*(33) = 4.24, *p* < .001. Further, this result was replicated in the nonseeing condition with participants being significantly more likely to detect a change at the Long SOA (*M* = .80, *SD* = .19) compared to the Short SOA (*M* = .68, *SD* = .19), *t*(33) = 6.00, *p* < .001. Finally, paired-samples *t* tests revealed that at the Long SOA there was no significant difference in accuracy between the seeing condition (*M* = .79, *SD* = .14) and nonseeing condition (*M* = .80, *SD* = .19), *t*(33) = .31, *p* =.755. This result was replicated at the Short SOA with no difference in accuracy between the seeing condition (*M* = .71, *SD* = .14) compared with the nonseeing condition (*M* = .68, *SD* = .19), *t*(33) = 1.60, *p = .*119.

These analyses confirmed that all participants were more accurate, and therefore more able to detect if a change had occurred, at the long SOA. However, they also revealed that SOA did not influence the extent to which MSA affected the ability of the participant to detect a change. Further, although all participants were more accurate at the Long SOA this did not influence the Validity × Condition interaction present in the Low Autistic Traits group. Therefore, the mental state attribution influenced the gaze cueing effect at SOAs associated with both bottom-up and top-down processing for individuals with low amounts of autistic traits.

## Discussion

The aim of the current study was to investigate whether autistic traits affect the extent to which mental state attributions can influence gaze cueing in neurotypical individuals. The results of this study very clearly demonstrate that autistic traits do influence whether mental states affect gaze cueing. Whilst both the High Autistic Traits group and the Low Autistic Traits group showed a robust gaze cueing effect, only the Low Autistic Traits group was influenced by the mental state of the cue-agent and showed a significantly reduced gaze cueing effect when they believed that the cue-agent was unable to see the target it was gazing towards. This effect was not observed in the High Autistic Traits group. Further, the mental state attribution was found to influence the gaze cueing effect for the Low Autistic Traits group at both the Long and Short SOA. This demonstrates that mental states, and theory of mind processes, not only influence our attentional processing in a top-down manner (as demonstrated at the Long SOA), but that these processes can also occur rapidly and automatically (as demonstrated at the Short SOA). This was observed only for participants in the Low Autistic Traits group. The results of this study are therefore three-fold: (1) mental state attributions can modulate gaze cueing; (2) individuals high in autistic traits do not use mental state information when processing gaze cues, but individuals low in autistic traits do; and (3) mental state attributions affect gaze cueing via both automatic attention cueing at short SOAs and conscious attention cueing at longer SOAs in individuals low in autistic traits.

The findings of this study therefore support the study hypothesis that autistic traits would affect the extent to which mental state attributions influence gaze cueing. Interestingly, both groups demonstrated a clear gaze cueing effect, and so both groups were clearly capable of following the gaze of another person. However, it is apparent that within our everyday lives we do not indiscriminately follow every eye movement to which we bear witness, thereby suggesting that we must engage some form of top-down processing to determine when it is most relevant to follow a gaze. In the case of this study via the attribution of a mental state. This study demonstrates that high amounts of autistic traits can lead to difficulties in attributing mental states to our social partners, which then leads to differences in gaze cueing behaviour in comparison to individuals with low amounts of autistic traits. The gaze of our social partners can act as a signal to important events and objects within our environment (Capozzi & Ristic, 2018). Therefore, if individuals do not have access to all of the nuanced information used to determine either when to follow a gaze or whose gaze to follow, then this has clear implications for how their attention is directed in the world around them. For those individuals in the High Autistic Traits group, the inability to automatically attribute a mental state to the gaze cue agent, and consequently discern when it was most appropriate to follow the gaze cue, therefore has clear implications for gaze following behaviour within their everyday lives.

Critically, the results of this study also support previous studies which have found that reflexive attentional orienting can be influenced by theory of mind processes (Baker et al., 2016; Furlanetto et al., 2016; Morgan et al., 2018). Whilst studies have suggested that mental state attributions do not always directly guide gaze cueing (Gobel et al., 2018; Kingstone et al., 2019). Nevertheless, the findings of this study contrast with recent research, which argues that the processes underlying reflexive gaze following are isolated from the influence of theory of mind or perspective taking processes (Millett et al., 2019; Cole & Millett, 2019). These studies draw their conclusions from experiments demonstrating that participants can show perspective taking effects even in the absence of a social partner (Wilson et al., 2017), or fail to take into account an obstacle blocking a partner’s view and respond as if the partner is able to ‘see’ through the barrier (as in line-of-sight barrier tasks; Cole et al., 2015). However, in contrast to these studies, our results replicated those found within our previous paper (Morgan et al., 2018) and clearly demonstrate that mental state attributions, and therefore theory of mind processes, influenced the extent to which individuals with low amounts of autistic traits followed the gaze of a social partner. Further, in line with the parameters outlined by Kuhn et al. (2018), the results of this study indicate that this mediation occurred automatically, as the attribution of the mental state transpired rapidly at an SOA associated with bottom-up reflexive processing and, secondly, the mediation occurred independently of any task goals. The participants in this experiment were directly informed that the gaze was nonpredictive and would not assist them with completion of the secondary change detection task. This study therefore demonstrates that not only do mental state attributions mediate gaze cueing but also that they can do so automatically.

These findings could potentially be due in part to the nature of the paradigm used in this study. Change detection tasks are more sensitive to behavioural changes (Santee & Egeth, 1982) and less susceptible to noise (Milliken & Tipper, 1998) than response time tasks. Further, these types of tasks are highly contingent on attention, such that participants are significantly more accurate at identifying changes that occur at a cued location (Smith & Schenk, 2008, 2010), which gave us the best possible chance of observing modulations of cueing that may not have been evident in reaction time tasks. Further, the manipulation used in this study was not only conveyed to participants via written instructions but was also reinforced through the use of videos. The videos allowed the participants to develop a further association between the colour of the sunglasses and the seeing or nonseeing condition. The use of these videos also encouraged the activation of theory of mind processes; recent research has indicated that even simply viewing another person engaging in an interaction (such as between the experimenter and cue-agent during the object identification task in the videos) is sufficient to generate changes associated with theory of mind processes at both a behavioural and cortical level (Gregory et al., 2015; Redcay & Schilbach, 2019). Priming the engagement of theory of mind processes prior to the beginning of the study may therefore have supported the rapid attribution of mental states to the cue-agent during the study.

Another consideration for the procedure of this study relates to the location of the manipulation that generated the mental state attribution. As discussed in our previous paper (Morgan et al., 2018), in this paradigm the mental state attribution is generated by directly manipulating the cue-agent, rather than the environment the cue-agent is situated in. This therefore allows the information about the gaze direction and the mental state of the cue-agent to be processed within the same spatial location, potentially facilitating the rapid processing, and combining, of these two differing pieces of information. This contrasts to other variations of perspective taking paradigms—for example, line-of-sight tasks—that instigate a mental state attribution by introducing a ‘barrier’ between the cue-agent and the target. In such instances the mental state manipulation is presented within the periphery of the participant’s attention, rather than aligning with the centrally presented cue-agent. The participant then must attend to and combine two spatially distinct pieces of information. This arguably leads to longer processing times as it adds an extra level of complexity to the paradigm (Wilson et al., 2017), which would not lend itself to the time courses associated with reflexive processing.

## Conclusion

The aim of this study was to investigate the impact of autistic traits on the mediation of the gaze cueing effect by mental state attributions, and whether these attributions would influence attentional orienting within a change detection task. In accordance with our previous findings, there was clear evidence that mental state attributions influenced whether a gaze cue was followed as participants were only likely to follow the gaze direction of a cue-agent if they believed the agent was able to see. However, critically, this effect was observed in the Low Autistic Traits group only. No evidence of this effect was observed in the High Autistic Traits group, the perceived mental state of the cue agent did not influence gaze cueing. Crucially, this result was found to extend beyond top-down processing at the long SOA and also influenced reflexive gaze cueing at the short SOA. These results are critical as they therefore demonstrate that autistic traits influence how mental state attributions modulate gaze following behaviour.

## Declarations

### Data availability of data and materials

The data that support the findings of this study are available in the Open Science Framework (osf.io/fjkhw/?view_only=d5711ebab1af4234ab7fcd49fd121654).
